# Comparison of *In Vitro* Cell Transformation Assay Using Murine Fibroblasts and Human Keratinocytes

**DOI:** 10.5487/TR.2008.24.1.037

**Published:** 2008-03-01

**Authors:** Jun-Ho Ahn, Sue Nie Park, Yung-Na Yum, Ji-Young Kim, Michael Lee

**Affiliations:** 16 Department of Biology, College of Natural Sciences, University of Incheon, 177 Dowha-dong, Nam-gu, Incheon, 402-749 Korea; 26 Division of Genetic Toxicology National Institute of Toxicological Research, Korea Food and Drug Administration, Seoul, 122-704 Korea; 36 Korea Institute of Toxicology, Korea Research Institute of Chemical Technology, Daejeon, 305-343 Korea

**Keywords:** Carcinogenicity, *In vitro* cell transformation assay, Murine fibroblasts, BALB/3T3, Human keratinocytes, HaCaT

## Abstract

The *in vitro* cell transformation assays (CTA) were performed using BALB/3T3 murine fibroblasts and HaCaT human keratinocytes in order to evaluate concordance between both *in vitro* CTAs and carcinogenicity with compounds differing in their genotoxic and carcinogenic potential. Six test articles were evaluated, two each from three classes of compounds: genotoxic carcinogens (2-amino-5-nitrophenol and 4-nitroquinoline-N-oxide), genotoxic noncarcinogens (8-hydroxyquinoline and benzyl alcohol), and nongenotoxic carcinogens (methyl carbamate and N-nitrosodiphenylamine). Any foci of size ≥ 2 mm regardless of invasiveness and piling was scored as positive in CTA with BALB/3T3. As expected, four carcinogens regardless of their genotoxicity had positive outcomes in two-stage CTA using BALB/3T3 cells. However, of the two genotoxic noncarcinogens, benzyl alcohol was positive CTA finding. We concluded that, of the 6 chemicals tested, the sensitivity for BALB/3T3 system was reasonably high, being 100%. The respective specificity for BALB/3T3 assay was 50%. We also investigated the correlation between results of BALB/3T3 assay and results from HaCaT assay in order to develop a reliable human cell transformation assay. However, evaluation of staining at later time points beyond the confluency stage did not yield further assessable data because most of HaCaT cells were detached after 2~3 days of confluency. Thus, after test article treatment, HaCaT cells were split before massive cell death began. In this modified protocol for this HaCaT system, growing attached colonies were counted instead of transformed foci 3 weeks since last subculture. Compared to BALB/3T3 assay, HaCaT assay showed moderate low sensitivity and high specificity. Despite these differences in specificity and sensitivity, both cell systems did exhibit same good concordance between *in vitro* CTA and rodent carcinogenicity findings (overall 83% concordant results). At present the major weakness of these *in vitro* CTA is lack of validation for regulatory acceptance and use. Thus, more controlled studies will be needed in order to be better able to assess and quantitatively estimate *in vitro* CTA data.

## INTRODUCTION

The conventional carcinogenicity test is the long-term rodent bioassay described in the OECD Test Guideline (TG) 451 (OECD, 1981). However, the rodent carcinogenicity assay is expensive and time-consuming. Thus, several *in vitro* alternatives to animal-based methods have been developed. The most commonly used *in vitro* test systems to predict carcinogenicity are the short-term *in vitro* assays such as the Ames test, chromosomal aberration test (CA) and mouse lymphoma assay (MLA), which are widely used to determine the genotoxic potential during preclinical drug development (Maurici *et al.*, 2005). The sensitivity of the genotoxicity assay from published database was high, but the specificity of the mammalian assays was poor (Lee *et al.*, 2002; Kirkland *et al.*, 2006). In fact, 75~90% of rodent non-carcinogens were positive in one or more of the assays, resulting in a high number of false positive results (Kirkland *et al.*, 2005). Also, non-genotoxic carcinogens, which exert their effect on cells through mechanisms devoid of direct interaction with DNA, were found to be negative in a variety of genotoxicity assays. The high false decision rate may be due to the multiple stages of carcinogenesis, and the multiple mechanisms.

The *in vitro* cell transformation assays (CTA), which mimic some stages of *in vivo* multistep carcinogenesis, have been proposed to identify possible non-genotoxic carcinogens in addition to genotoxic carcinogens. Compared to *in vivo* based-assays, *in vitro* CTA tests are fast, cost efficient and provide a means for initial screening of carcinogenic potential. A positive result is where there is a significant increase in the number of morphologically transformed foci (Combes *et al.*, 1999). Since the *in vitro* CTA using BALB/3T3 cells was developed by Kakunaga (1973), an analysis of data for many chemicals obtained by using an optimised BALB/3T3 assay protocol, has been published by Matthews *et al.* (Matthews, 1993a, b, c). A new protocol has been further developed to improve transformation frequency, and especially to shorten the length of the assay (Tsuchiya and Umeda, 1995; Tsuchiya *et al.*, 1999).

Using the improved method (Tsuchiya and Umeda, 1995) of *in vitro* CTA suggested by IARC/NCI/EPA Working Group (1985), we have evaluated its ability to predict rodent carcinogenicity and compared *in vitro* CTA using HaCaT human keratinocytes with the conventional *in vitro* CTA results using BALB/3T3 murine fibroblasts. The *in vitro* CTA was performed on six test articles: two genotoxic rodent carcinogen (2-amino-5-nitrophenol and 4-nitroquinoline-N-oxide), two genotoxic non-carcinogen (8-hydroxyquinoline and benzyl alcohol), and two non-genotoxic carcinogen (methyl carbamate and N-nitrodiphenylamine). Overall the data indicate that a reasonably good concordance between *in vitro* transformation and *in vivo* carcinogenicity was obtained in both cell systems of BALB/3T3 and HaCaT, although the *in vitro* CTA using human cell systems should be further developed and validated for regulatory use.

## MATERIALS AND METHODS

***Cell lines and cell culture.*** BALB/3T3 A31 cells (murine fibroblasts) were purchased from American Type Culture Collection (ATCC, Manassas, VA) while HaCaT cells (human keratinocytes) were obtained from Dr. S. N. Park (National Institute of Toxicological Research/KFDA, Seoul, Korea). As previously described (Lee *et al.*, 2002; Piao *et al.*, 2006), cells were grown in Dulbecco's modified Eagle's medium (DMEM, GIBCO-Invitrogen, Carlsbad, CA) supplemented with 10% fetal bovine serum (FBS, GIBCO-Invitrogen), penicillin/streptomycin, and glutamine. Only 60~70% confluent cells were subcultured. They were cultured in a humidified $CO_2$ incubator under 5% $CO_2$ and 95% $O_2$. DMEM/F12 medium (GIBCO-BRL) and ITES (20 μg/ml bovine pancreas insulin, 200 μg/ml human transferrin, 12.2 μg/ml ethanolamine, and 0.034 μg/ml sodium selenite) (Sigma, St Louis, MO) were used for transformation assay.

***Materials.*** The test articles used for *in vitro* cell transformation assay in this study were: 2-amino-5-nitrophenol (CAS# 121-88-0), 4-nitroquinoline-N-oxide (CAS# 56-57-5), 8-hydroxyquinoline (CAS# 148-24-3), benzyl alcohol (CAS# 100-51-6), methyl carbamate (CAS# 598-55-0), and N-nitrosodiphenylamine (CAS# 86-30-6). All were obtained from Sigma-Aldrich (St. Louis, MO). Giemsa solution and crystal violet were also purchased from Sigma-Aldrich.

***Colony forming efficiency.*** The assay used to measure cellular ability to form colonies was as described in OECD detailed review paper (OECD, 2007). Cells were seeded at a density of 200 cells/60 mm petri dish. After 24 h, test articles were added at different concentrations. After 72-h exposure the culture medium was replaced with fresh medium and changed twice a week. Seven days later, cells were washed with 1 × PBS, fixed with methanol and stained with 5% Giemsa solution for counting the number of colonies.

***WST-1 assay.*** The cell proliferation reagent WST-1 was used for the quantitative determination of cellular proliferation (Roche Molecular Biochemicals, Germany). For proliferation assays, cells were plated in quadruplicates into 96-well microliter plates (Costar, Cambridge, MA) at $5 \times 10^3$ cells/well and cultivated for 24 h, prior to addition of each test article. The cells were then treated with test articles at 37°C in a humidified 5% $CO_2$/95% air incubator. After incubation for 3 days, the cells were incubated for additional 4 h in the presence of WST-1 labeling mixture (10 ml per well). The absorbance of the samples against a background control (medium alone) as a blank, was measured at 450 nm using a microliter plate (ELISA) reader (Molecular Devices, Sunnyvale CA).

***One-stage in vitro cell transformation assay.*** The standard *in vitro* cell transformation assay was performed according to the method suggested by IARC/NCI/EPA Working Group (1985). Actively growing cells are seeded at a density of $10^4$ cells/60-mm dish (10 dishes for each treatment) in 5 ml of culture medium.

After 24 hr incubation subsequent to plating cells, culture medium is removed from each plate and replaced with medium containing a test article. Cells are exposed to the test article for 72 hr. The medium is replaced with fresh medium and changed twice a week during the following 5 weeks. At the end of incubation the medium is removed and the cells are rinsed with saline, fixed in methanol, and stained with 5% aqueous Giemsa for scoring focus formation. Morphological transformation was determined under a dissecting microscope. Only densely stained multilayer foci with criss-crossing of the cells at their periphery were scored as transformed foci (Keshava, 2000).

***Two-stage in vitro cell transformation assay.*** Actively growing cells are seeded at a density of $10^4$ cells/60-mm dish (10 dishes for each treatment) in 5 ml of culture medium. After 24 hr incubation subsequent to plating cells, culture medium was removed from each plate and replaced with medium containing a test article. Four days after, the culture medium was replaced by fresh medium containing phorbol 12-myristate 13-acetate (PMA). Promotion was continued by replacing the medium containing PMA for 2 weeks. DMEM medium supplemented with insulin-transferrin-ethanolamine-sodium selenite (ITES) plus 2% FCS, which was found to enhance the frequency of focus formation (Tsuchiya and Umeda, 1995), was used during the period of expression of transformed foci (Kajiwara and Ajimi, 2003). Five to 6 weeks after the first test article treatment, cultures were fixed in methanol and stained with a 5% Giemsa solution. Morphological transformation was determined under a dissecting microscope. In case of HaCaT human keratinocytes, most of the cells were detached after 2~3 days of confluency. Thus, HaCaT cells were re-seeded at a density of $10^4$ cells/60-mm dish at 2 weeks and 3 week after test article treatment. Growing attached colonies were counted instead of transformed foci 3 weeks since last subculture.

## RESULTS

***Selection of test articles.*** The *in vitro* cell transformation assays (CTA) were performed in BALB/3T3 murine fibroblasts and HaCaT human keratinocytes on six test substances: two genotoxic carcinogens (2-amino-5-nitrophenol and 4-nitroquinoline-N-oxide), two genotoxic noncarcinogens (8-hydroxyquinoline and benzyl alcohol), and two nongenotoxic carcinogens (methyl carbamate and N-nitrosodiphenylamine). Information on the carcinogenicity and genotoxicity of each test substance was obtained from the previous published reports (Table 1).

***Measurement of cytotoxicity.*** The dose range-finding assays were performed to select the concentration that induced an inhibition of cell growth by more than 80%. Two independent studies were performed to assess cytotoxicity, the acute WST-1 colorimetric test and the colony-formation efficiency (CFE) test. In both cell lines of BALB/3T3 and HaCaT, CFE test appeared to be more sensitive to test article treatment than the WST-1 colorimetric test (Table 2). Especially, in either 4-nitroquinoline- or N-nitrosodiphenylamine-treated cells, significant growth inhibition could be observed in CFE

**Table 1.** Published genotoxicity and carcinogenicity results for the test articles

| Test articles | CAS No. | Genotoxicity | | | 2-year rodent carcinogenicity |
|---|---|---|---|---|---|
| | | Ames | MLA | *In vitro* CA | |
| Genotoxic carcinogen | | | | | |
| 2-Amino-5-nitrophenol | 121-88-0 | +(1) | +(2) | +(3) | +(4) |
| 4-Nitroquinoline-*N*-oxide | 56-57-5 | +(1) | +(2) | +(3) | +(5) |
| Genotoxic non-carcinogen | | | | | |
| 8-Hydroxyquinoline | 148-24-3 | +(4) | +(2) | +(6) | -(4) |
| Benzyl alcohol | 100-51-6 | -(1) | +(2) | +(7) | -(4) |
| Non-genotoxic carcinogen | | | | | |
| Methyl carbamate | 598-55-0 | -(1) | -(2) | -(7) | +(4) |
| *N*-Nitrosodiphenylamine | 86-30-6 | -(1) | -(2) | -(8) | +(4) |

+, positive response; -, negative response; Ames, reverse bacterial mutation assay in *Salmonella typhimurium; in vitro* CA, in vitro chromosomal aberration assay; MLA, mouse lymphoma assay.
(1) Gold and Zeiger (1997) Handbook of carcinogenic potency and enotoxicity databases. pp. 687; (2) Mitchell *et al.* (1997) Mutat. Res. 394:177; (3) Ishidate Jr. *et al.* (1986) Mutat. Res. 195:151; (4) Gold, L.S. (2004). The carcinogenic potency project. http://potency.berkeley.edu/cpdb.html; (5) Nesnow *et al.* (1986) Mutat. Res. 185:1; (6) Armstrong *et al.* (1992) Mutat. Res. 265:45; (7) NTP, NTP website at http://ntp-server.niehs.nih.gov.; (8) Loveday *et al.* (1990) Environ, Mol. Mutagen. 16;272.

**Table 2.** Comparison of cytotoxicity measurements using colony-formation efficiency and WST-1 colorimetric assay

| Test articles | Balb/3T3 | | HaCaT | |
|---|---|---|---|---|
| | CFE ($LC_{50}$) | WST-1 ($LC_{50}$) | CFE ($LC_{50}$) | WST-1 ($LC_{50}$) |
| 2-Amino-5-nitrophenol | $LC_{90}$ < 0.05 mM | 0.8 mM | 0.1~0.2 mM | 1.1 mM |
| 4-Nitroquinoline | 1 ng/ml | > 10 ng/ml | 1 ng/ml | > 50 ng/ml |
| 8-Hydroxyquinoline | $LC_{90} \approx 1$ μM | 1.6 μM | 1~2 μM | > 5 μM |
| Benzyl alcohol | 3 mM | > 10 mM | 7~10 mM | 10 mM |
| Methyl carbamate | 60 mM | 150~170 mM | 100 mM | 150 mM |
| *N*-Nitrosodiphenylamine | 0.05 mM~0.1 mM | > 0.6 mM | 0.1 mM~0.2 mM | > 0.6 mM |

CFE, Colony-formation efficiency. $LC_{50}$, The lethal concentration that kills 50% of cells. $LC_{90}$, The lethal concentration that kills 90% of cells.

test while the cytotoxicities caused by both test articles were not evident even at the highest concentration in WST-1 colorimetric test. Also in case of other four test articles, cytotoxicity was observed at much lower concentration in CFE assay. Thus, dose ranges for *in vitro* CTA were determined through CFE test results.

***In vitro cell transformation assay using BALB/3T3 murine fibroblasts.*** *In vitro* two-stage cell transformation assay using BALB/3T3 cells has been recognized as being a useful experimental method in which carcinogenic events can be studied in relation to both initiation and promotion (Sakai and Sato, 1989; Tsuchiya *et al.*, 1999). Thus, in addition to the conventional one-stage *in vitro* CTA well established for routine carcinogen testing (IARC/NCI/EPA Working Group, 1985), two-stage CTA was also performed. In the one-stage CTA, the mean number of transformed foci of the vehicle control was 0.3~0.4 per dish while, in the two-stage CTA with PMA promotion, the mean number of transformed foci per dish was 0.9~1.4 (Table 3) and the percentage of dishes with foci was 60.0% (12 of 20 dishes, data not shown). In general, the two-stage CTA appeared to be more sensitive to test article treatment than the one-stage CTA. The two-stage CTA clearly enhanced the frequency of transformed focus forma-

**Table 3.** *In vitro* cell transformation using Balb/3T3 murine fibroblasts

| Chemical | | Conc | No. of foci/plate | Chemical | | Conc | No. of foci/plate |
|---|---|---|---|---|---|---|---|
| 2-Amino-5-nitrophenol (μM) | One-stage | 0 | 0.4 ± 0.5 | 4-Nitroquinoline-*N*-oxide (ng/ml) | One-stage | 0 | 0.4 ± 0.5 |
| | | 25 | 1.1 ± 0.6 | | | 0.5 | 0.4 ± 0.5 |
| | | 50 | 1.8 ± 1.5* | | | 1 | 1.1 ± 0.6 |
| | | 75 | 2.7 ± 1.1** | | | 2 | 1.9 ± 0.8** |
| | Two-stage | 0 | 1.4 ± 0.9 | | Two-stage | 0 | 1.4 ± 0.9 |
| | | 25 | 2.7 ± 1.0 | | | 0.5 | 2.7 ± 1.0 |
| | | 50 | 4.7 ± 1.3** | | | 1 | 4.3 ± 1.4** |
| | | 75 | 4.0 ± 1.8** | | | 2 | 5.6 ± 1.7** |
| 8-Hydroxy-quinoline (μM) | One-stage | 0 | 0.4 ± 0.5 | Benzyl alcohol (mM) | One-stage | 0 | 0.3 ± 0.5 |
| | | 0.5 | 0.3 ± 0.5 | | | 3 | 0.4 ± 0.5 |
| | | 0.75 | 0.1 ± 0.4 | | | 5 | 0.7 ± 0.9 |
| | | 1 | 0.4 ± 0.5 | | | 7 | 1.9 ± 1.0** |
| | Two-stage | 0 | 1.4 ± 0.9 | | Two-stage | 0 | 0.9 ± 0.9 |
| | | 0.5 | 0.9 ± 0.6 | | | 3 | 2.0 ± 0.7 |
| | | 0.75 | 1.0 ± 0.9 | | | 5 | 1.8 ± 1.4 |
| | | 1 | 1.1 ± 0.6 | | | 7 | 2.6 ± 1.2** |
| Methyl carbamate (mM) | One-stage | 0 | 0.3 ± 0.5 | *N*-Nitroso-diphenylamine (μM) | One-stage | 0 | 0.3 ± 0.5 |
| | | 60 | 1.0 ± 0.5* | | | 50 | 0.7 ± 0.7 |
| | | 80 | 1.0 ± 0.5* | | | 75 | 0.9 ± 0.8 |
| | | 100 | 1.5 ± 0.5** | | | 100 | 0.9 ± 0.7 |
| | Two-stage | 0 | 0.9 ± 0.8 | | Two-stage | 0 | 0.9 ± 0.8 |
| | | 60 | 1.7 ± 1.0 | | | 50 | 1.4 ± 0.5 |
| | | 80 | 2.4 ± 0.9** | | | 75 | 1.7 ± 1.1 |
| | | 100 | 2.4 ± 0.9** | | | 100 | 2.3 ± 0.7** |

*Significantly different from the control at $P < 0.05$. **Significantly different from the control at $P < 0.01$.

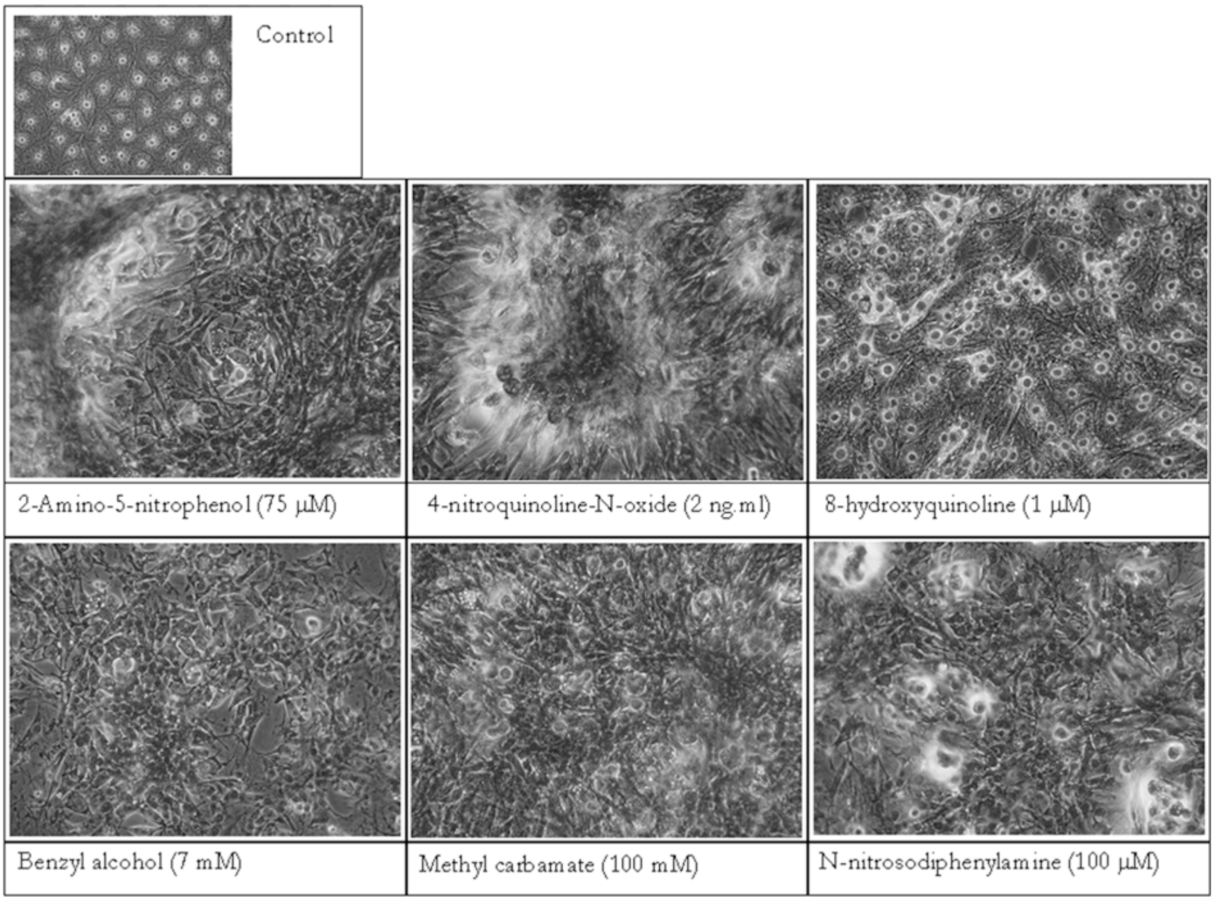

**Fig. 1.** Morphological changes of Balb/3T3 cells after the addition of test articles. (A) Balb/3T3 control at 6 weeks; (B) Balb/3T3 with 75 μM 2-Amino-nitrophenol with PMA at 6 weeks; (C) Balb/3T3 with 2 ng/ml 4-nitroquinoline with PMA at 6 weeks; (D) Balb/3T3 with 1 μM 8-hydroxyquinoline with PMA at 6 weeks; (E) Balb/3T3 with 7 mM benzyl alcohol with PMA at 6 weeks; (F) Balb/3T3 with 100 mM methyl carbamate with PMA at 6 weeks; (G) Balb/3T3 with 100 μM N-nitrosodiphenylamine with PMA at 6 weeks.

tion. As expected, two genotoxic carcinogens and two non-genotoxic carcinogens had positive outcomes in two-stage CTA although, of the nongenotoxic carcinogens, one (N-nitrosodiphenylamine) was negative finding in one-stage CTA. Of the two genotoxic noncarcinogens, benzyl alcohol was positive CTA finding. In particular, the representative photomicrographs of transformed foci induced by six test articles are shown in Fig. 1.

***In vitro* cell transformation assay using HaCaT human keratinocytes.** Upon reaching confluence in culture HaCaT cells started to detach and most of the cells were detached after 2~3 days of confluency. Detached cells did not show signs of further growth. Thus, evaluation of staining at later time points beyond the confluency stage did not yield further assessable data since cells then start to die. Therefore, after test article treatment, HaCaT cells were split before massive cell death began. Instead of transformed foci, growing attached colonies were counted 3 weeks since last subculture (Table 4). By this criteria, we found that, using two-stage CTA, all of two genotoxic carcinogens had positive CTA outcomes while, of the two nongenotoxic carcinogens, N-nitrosodiphenylamine was negative CTA finding. All of the two genotoxic noncarcinogens had negative CTA finding.

***Relationship between outcome of CTA using BALB/3T3 and HaCaT cells.*** The reported carcinogenicity results for each test article are presented in Table 1. With our limited *in vitro* CTA data-set, an investigation into the predictive value of *in vitro* CTA tests for determining the carcinogenicity was carried out (Table 5). Statistical analyses include sensitivity (the ability of a test to predict carcinogenicity), specificity (the ability of a test to predict non-carcinogenicity), positive predictivity

**Table 4.** *In vitro* cell transformation assay using HaCaT human keratinocytes

| Chemicals | Conc. | No. of foci/plate | Chemicals | Conc. | No. of foci/plate |
|---|---|---|---|---|---|
| 2-Amino-5-nitrophenol (μM) | 0 | Negative | 4-Nitroquinoline-*N*-oxide (ng/ml) | 0 | Negative |
| | 100 | Positive | | 0.5 | Positive |
| | 150 | Positive | | 1 | Positive |
| | 200 | Positive | | 2 | Positive |
| 8-Hydroxyquinoline (μM) | 0 | Negative | Benzyl alcohol (mM) | 0 | Negative |
| | 1 | Negative | | 5 | Negative |
| | 1.5 | Negative | | 7 | Negative |
| | 2 | Negative | | 10 | Negative |
| Methyl carbamate (mM) | 0 | Negative | *N*-Nitroso-diphenylamine (μM) | 0 | Negative |
| | 60 | Positive | | 100 | Negative |
| | 100 | Positive | | 150 | Negative |
| | 140 | Positive | | 200 | Negative |

**Table 5.** Relationship between outcome of single toxicology assay and rodent carcinogenicity assay

| | | Balb/3T3 murine fibroblasts | | HaCaT human keratinocytes | |
|---|---|---|---|---|---|
| | | - | + | - | + |
| Carcinogenicity | - | 1 | 1 | 2 | 0 |
| | + | 0 | 4 | 1 | 3 |
| | Total | 1 | 5 | 3 | 3 |
| Sensitivity[a] | | 1.0 | | 0.75 | |
| Specificity[b] | | 0.5 | | 1.0 | |
| Negative predictivity[c] | | 1.0 | | 0.67 | |
| Positive predictivity[d] | | 0.8 | | 1.0 | |
| Concordance[e] | | 0.83 | | 0.83 | |

The carcinogenicity of chemical substances as tested in rodents (+, carcinogen; -, not carcinogen) was compared with the results from each genotoxicity assays.
[a]Proportion of carcinogens positive in a particular genotoxicity test.
[b]Proportion of non-carcinogens negative in a particular genotoxicity test.
[c]Proportion of genotoxicity test negatives that are non-carcinogens.
[d]Proportion of genotoxicity test positives that are carcinogens.
[e]Proportion of qualitative agreements between carcinogenicity results and a particular genotoxicity results.

(the proportion of chemicals accurately predicted to be carcinogens by a particular test), negative predictivity (the proportion chemicals accurately predicted to be non-carcinogens by a particular test) and concordance (the proportion of chemicals correctly identified by a particular test). The *in vitro* CTA with BALB/3T3 cells has high sensitivity and low specificity and, vice versa, for *in vitro* CTA with HaCaT cells sensitivity is moderate low and specificity high. However, both cell lines did exhibit same concordance between *in vitro* CTA and rodent carcinogenicity findings (1/6 cases with discrepancies and overall 83% concordant results).

## DISCUSSION

The *in vitro* cell transformation has been known to be a multistage process, which closely mimics *in vivo* carcinogenesis (LeBoeuf *et al.*, 1999). Since the *in vitro* CTA can be responsive to chemicals acting via both genotoxic and non-genotoxic mechanisms, which cannot be detected by genotoxicity testing, (Nakajima *et al.*, 2005), it is increasingly used as a biomarker of exposure to putative carcinogens. Among the several types of cell transformation assays, we used the foci assay using the BALB/3T3 murine fibroblasts because of its good reproducibility (Kakunaga and Yamasaki, 1985) and inter-laboratory validation of experimental protocol (OECD, 2007). In foci assay, cells are exposed to a test article in culture, and then assessed for their ability to form foci of morphologically transformed cells against a confluent monolayer of cells. According to the Keshava's suggestion (Keshava, 2000), any foci of size ≥ 2 mm regardless of invasiveness and piling was scored as positive although BALB/3T3-transformed foci can be characterized based on several properties such as size, invasiveness and piling properties (IARC/NCI/EPA Working Group, 1985; Landolph, 1985). In this study, the *in vitro* CTA was performed with 6 test articles whose genotoxicities and carcinogenicity are known in a wide range of test systems. As expected, a reasonably good concordance between BALB/3T3 assay and *in vivo* carcinogenicity was obtained for carcinogens, regardless of genotoxicity. However, of the two genotoxic noncarcinogens, benzyl alcohol was positive CTA finding, indicating that the respective specificity for Balb/3T3 assay was 50%.

The ultimate goal of the rodent carcinogenicity assay is to determine the risk of human exposure to potential carcinogens. Due to species differences such as xenobiotics metabolism, and frequencies of spontaneous and induced transformation of cells in culture between rodents and humans, assay system with rodent-derived cells could lead to false decision for cancer development in human. Therefore, the development of *in vitro* CTA using human cell system is very important in prediction of human carcinogenicity. Since no test system with immortalized human cells has yet been standardized, we validated the *in vitro* CTA with HaCaT human cells using 6 test articles through comparison with BALB/3T3 system. The HaCaT cell line was derived by spontaneous immortalization of normal human keratinocytes (Boukamp *et al.*, 1988, 1997) and was able to be transformed to tumorigenic variants by *ras* oncogene transfection (Boukamp *et al.*, 1995; Fusenig and Boukamp, 1998). During the *in vitro* CTA, we noted that most of the HaCaT cells were detached after 2~3 days of confluency. Thus, HaCaT cells needed to be maintained at less than 85% confluency in order to preserve their attached states. To avoid this problem, after test article treatment, HaCaT cells were split before massive cell death began. Instead of transformed foci, growing attached colonies were then counted 3 weeks since last subculture. In fact, there are no defined *in vitro* criteria for distinguishing between tumorigenic and non-tumorigenic HaCaT cells. In addition, despite many reports regarding HaCaT system (Fusenig and Boukamp, 1998) in human cell transformation assays, HaCaT cells have not been thus far transformed to focus-forming cells after carcinogen treatment in a manner similar to that described for the rodent cell transformation assays. Our results showed that, of the two nongenotoxic carcinogens, N-nitrosodiphenylamine was negative CTA finding. Compared to BALB/3T3 system, the modified protocol for this HaCaT system had a lower sensitivity for this class of chemical and a higher specificity for genotoxic non-carcinogens. More controlled studies are needed in order to be better able to assess and quantitatively estimate *in vitro* CTA data in HaCaT system.

For determination of cytotoxicity for each test article, two independent studies, WST-1 colorimetric method and the colony-formation efficiency (CFE) test, were performed. Actually, CFE test provides the most unequivocal quantitative measurement of survival. However, CFE test is time-consuming and labor-intensive, and become impractical especially when many compounds must be screened rapidly. The colorimetric method, which are performed in 96-well plates and assessed using an ELISA reader, is faster and easier to perform, and it has a higher statistical power. Thus, we have investigated the correlation between results of the colorimetric method and results from the CFE if colorimetric assays could be substituted for CFE test. In either 4-nitroquinoline- or N-nitrosodiphenylamine-treated cells, significant growth inhibition could be observed in CFE test while the cytotoxicities were not evident even at the highest concentration in WST-1 colorimetric test. These results indicate that the WST-1-based colorimetric assay is considerably less sensitive than CFE test.

There is accumulating evidence that the *in vitro* CTA is a reliable test system because it detected transformed foci in good agreement with the results of rodent carcinogenicity (LeBoeuf *et al.*, 1999). An analysis of *in vitro* CTA data for 161 chemicals including 84 carcinogens and 77 non-carcinogens by Matthews *et al.* (1993a, b, c) revealed that the overall concordance was 71%, with a sensitivity of 80% and a specificity of 60%. Despite these reported results, the use of *in vitro* CTA is relatively low due to the difficulty of technical skills, the lack standardization and validation for regulatory acceptance and use. In addition, the results from the collaborative trials have revealed problems in inter-laboratory and intra-laboratory reproducibility because many kinds of protocols have been used in their studies (Muller *et al.*, 1993). Thus, further research is required to standardize the currently available systems before they can be considered ready to substitute for rodent carcinogenicity assay.

## ACKNOWLEDGMENTS

This research was supported by a grant (07142NTP600) from Korea Food & Drug Administration in 2007.
